# American Edition of Nothnagel’s Encyclopedia

**Published:** 1902-01

**Authors:** 


American Edition of Nothnagel's Encyclopedia.—Typhoid and Typhus Fevers. By Dr. H. Curschmann, of Leipzig. Edited, with additions, by William Osler, M. D., Professor of the Principles and Practice of Medicine, Johns-Hopkins University. Handsome octavo of 646 pages, illustrated, including a number of valuable temperature charts and two full-page colored plates. Philadelphia and London: W. B. Saunders & Co. 1901. Cloth, $5.00 net; sheep or half Morocco, $6.00 net.

In his preface to this volume Dr. Osler says "the original edition is recognized by all special students of typhoid fever as the standard authority on the subject." This statement has at least the weight of probability in its support, for here are almost five hundred pages devoted solely to a discusson of typhoid fever, written by the eminent H. Curschmann, edited by the no less distinguished

William Osler of this country, and translated into English by Alfred Stengle of the University of Pennsylvania. No work of the kind has ever been offered to the profession with higher recommendation.

In preparing the American edition Dr. Osler has made important additions to many of the chapters. In doing so he has freely consulted American literature. For example, Mallory's work on the pathology of the disease, Thayer's study of the state of the blood, and W. W. Keen's monograph on the surgical complications have all been utilized. R.

